# TRPM5, a taste-signaling transient receptor potential ion-channel, is a ubiquitous signaling component in chemosensory cells

**DOI:** 10.1186/1471-2202-8-49

**Published:** 2007-07-04

**Authors:** Silke Kaske, Gabriele Krasteva, Peter König, Wolfgang Kummer, Thomas Hofmann, Thomas Gudermann, Vladimir Chubanov

**Affiliations:** 1 Philipps-University Marburg, Institute of Pharmacology and Toxicology, Karl-von-Frisch-Straße 1, 35032 Marburg, Germany; 2 Justus-Liebig-University Gießen, Institute of Anatomy und Cell Biology, Aulweg 123, 35385 Giessen, Germany; 3 University of Lübeck, Institute of Anatomy, Ratzeburger Alle 160, 23538 Lübeck, Germany

## Abstract

**Background:**

A growing number of TRP channels have been identified as key players in the sensation of smell, temperature, mechanical forces and taste. TRPM5 is known to be abundantly expressed in taste receptor cells where it participates in sweet, amino acid and bitter perception. A role of TRPM5 in other sensory systems, however, has not been studied so far.

**Results:**

Here, we systematically investigated the expression of TRPM5 in rat and mouse tissues. Apart from taste buds, where we found TRPM5 to be predominantly localized on the basolateral surface of taste receptor cells, TRPM5 immunoreactivity was seen in other chemosensory organs – the main olfactory epithelium and the vomeronasal organ. Most strikingly, we found solitary TRPM5-enriched epithelial cells in all parts of the respiratory and gastrointestinal tract. Based on their tissue distribution, the low cell density, morphological features and co-immunostaining with different epithelial markers, we identified these cells as brush cells (also known as tuft, fibrillovesicular, multivesicular or caveolated cells). In terms of morphological characteristics, brush cells resemble taste receptor cells, while their origin and biological role are still under intensive debate.

**Conclusion:**

We consider TRPM5 to be an intrinsic signaling component of mammalian chemosensory organs, and provide evidence for brush cells being an important cellular correlate in the periphery.

## Background

Transient receptor potential (TRP) proteins form a large gene family of ion channels characterized by distinct activation mechanisms and biophysical properties. By sequence homology, members of the family fall into six subfamilies (TRPC, TRPV, TRPM, TRPML, TRPP, and TRPA). There is mounting evidence that TRP channels are involved in thermosensation, mechanosensation, smell and taste. A subset of TRP channels, called 'thermo-TRPs' (TRPV1-TRPV4, TRPA1 and TRPM8), have been found to be highly temperature dependent and are directly involved in heat and cold sensation in the peripheral nervous system [1]. Several TRP channels are mechanosensitive or activated by hypotonic challenge (TRPV4, TRPA1, TRPM3, PKD1 and TRPP2) [2]. TRPC2 is specifically expressed in the rodent sensory epithelium of the vomeronasal organ (VNO) where it plays a critical role in signaling processes triggered by pheromones [3,4]. More recently, evidence was obtained for a critical role of TRP channels in taste perception. Thus, PKD2L1 (TRPP2) and PKD1L3 are co-expressed in a subset of taste receptor cells (TRC) which are responsible for the detection of sour tastants [5,6]. TRPM5 was found to be essential for sweet, bitter and umami taste perception. TRPM5 is immunolocalized in a subset of TRC, [7,8] and two independently generated TRPM5 knock-out mouse models display diminished sweet, bitter and umami perception [9,10]. Interestingly, the sensitivity of TRPM5 to temperature was suggested to be the molecular mechanism underlying the psychophysical phenomenon of "thermal taste", i.e. enhanced sweetness perception with increasing temperature [11]. Studies of the biophysical properties of TRPM5 in heterologous expression systems by us and other groups revealed a monovalent-selective cation channel, directly gated by intracellular calcium, which rises upon stimulation of guanine nucleotide-binding regulatory protein (G protein)-coupled receptors (GPCR) linked to phospholipases type C (PLC). Furthermore, TRPM5 is regulated by voltage and phosphatidylinositol bisphosphate (PIP_2_) [10,12-14]. TRPM4, the closest homologue of TRPM5, displays a similar activation mechanism and channel properties [14,15]. TRPM4 and TRPM5 have been proposed as molecular candidates for calcium-activated nonselective (CAN) cation channels observed in many excitable and non-excitable cells [16]. CAN channels are assumed to be involved in membrane depolarization and, consequently, in the regulation of the intracellular calcium concentration.

Mechanistically, however, the role of TRPM5 in TRC is poorly understood. Taste buds consist of four different cell types (I-IV). Type II taste receptor cells are assumed to be directly involved in sweet, bitter and umami perception [17]. Taste compounds stimulate GPCRs for sweet and amino acids (T1R) [18,19] and bitter (T2R) [20,21] resulting in activation of the G-protein gustuducin (Gα_gust_). Consequently, calcium is released from internal stores via activation of PLCβ2 and subsequent inositol-1,4,5-trisphosphate (IP_3_) binding to IP_3 _receptor type III (IP_3_RIII). Elevated calcium levels activate TRPM5 leading to depolarization of the plasma membrane due to influx of Na^+^. The molecular events following TRPM5 activation are still under debate. Recently, gap junction hemichannels were proposed to be involved downstream of TRPM5 activation in taste signal transduction [22]. It is also unknown whether TRPM5 is associated with T1R/T2R and Gα_gust _as one functional unit due to sub-cellular compartmentalization, or whether TRPM5 represents an independent signaling component localized in distinct and specialized subcellular compartments of TRC.

It is assumed that the gastrointestinal and the respiratory tracts have the ability to analyze the composition of their luminal content in order to adequately respond to toxins and irritants. Thus, signaling molecules enriched in TRC have also been identified in populations of solitary epithelial cells in organs other than taste buds. For example, Gα_gust _was found in brush cells of the gastrointestinal tract and in pancreatic duct cells [23,24]. Bitter taste receptors, T2Rs, Gα_gust_, PLCβ2 and the IP_3_III receptor were immunolocalized in solitary chemosensory cells (SCC) in the larynx and respiratory epithelium [25-28]. Gα_gust _was found to be expressed in L type enteroendocrine cells in the human colon [29]. These observations support the notion that a population of specialized epithelial cells is present in different organs and may constitute part of a diffuse chemosensory system [30,31]. At present, common functional, morphological and biochemical criteria defining this putative cell system or these systems are not available and are the subject of intensive investigation.

Here we report that TRPM5 is highly abundant in rodent chemosensory organs including taste buds, the olfactory epithelium and the VNO, as well as in a subset of solitary cells distributed throughout the epithelia of the respiratory system and the gastrointestinal tract. The latter cells display morphological and biochemical cell features of brush cells (also known as tuft, fibrillovesicular, multivesicular or caveolated cells). Collectively, our data suggest that TRPM5 expression defines a population of specialized chemosensory cells and, thus, may offer a new point of entry to dissect molecular mechanisms involved in the processing of environmental cues by the respiratory system and the gastrointestinal tract.

## Results

### Localization of TRPM5 on the basolateral surface of taste receptor cells

**Figure 1:**
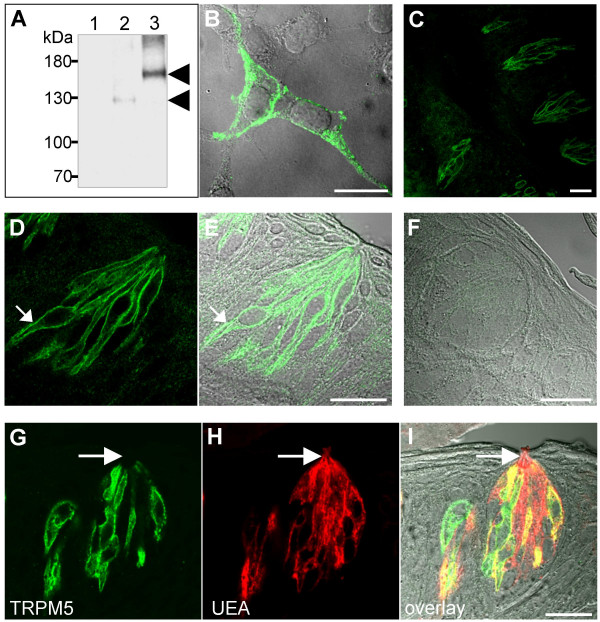
**Immunolocalization of TRPM5 in taste buds of mouse papillae vallatae**. The TRPM5-specific antibody Ab-321 recognizes TRPM5 transiently expressed in HEK 293 cells. (**A**) Western blot analysis of total lysates of mock- (line 1), TRPM5- (line 2) and YFP-TRPM5- (line 3) transfected HEK 293 cells. Positions of TRPM5- and YFP-TRPM5-specific bands are indicated by arrowheads. (**B**) Overlay of confocal and corresponding differential interference contrast (DIC) images of immunofluorescence staining of HEK 293 cells transiently expressing wild-type TRPM5. (**C-E**) Immunohistochemistry of taste buds using a TRPM5-specific antibody at lower (C) and higher magnifications (D, E). Notably, TRPM5 is predominantly localized on the basolateral surface of cells (indicated by the arrow in (D, E)). (**F**) Lack of TRPM5 immunoreactivity in taste receptors after preincubation of the TRPM5-specific antibody with the immunization peptide. (**G-I**) Fluorescent labeling of taste buds with the TRPM5-specific antibody (E) and the UEA lectin (H) and overlay of both with corresponding DIC image (I). Note that only a subset of cells in the taste bud is enriched in TRPM5. In the TRPM5-positive cells, TRPM5 is mainly localized on the basolateral cell surface and absent in microvilli (as indicated by an arrow (G, H, I)). Scale bars are 20 μm each.

To elucidate a biological role of TRPM5 we set out to study its expression pattern in rodent tissues using an immunohistochemical approach. To this end, we generated a polyclonal anti-TRPM5 antibody (AB-321) and tested its specificity in HEK 293 cells transiently transfected with the cDNA of mouse TRPM5. In Western blots of whole-cell lysates obtained from HEK 293 cells expressing either TRPM5 or YFP-TRPM5, the anti-TRPM5 antibody detected protein bands of expected sizes (~130 kDa and ~160 kDa, respectively) (Figure 1A). The immunoreactivity was abolished by preincubation of the antibody with the corresponding immunization peptide. Next, we tested the AB-321 antibody by indirect immunofluorescence staining of HEK 293 cells (Figure 1B). In contrast to untransfected cells, TRPM5-expressing cells displayed a fluorescent signal, which could be completely blocked by the corresponding immunization peptide. Of note, the subcellular distribution of TRPM5 immunoreactivity was similar to that observed by imaging of living HEK 293 cells expressing TRPM5 fused to a YFP tag (data not shown). To summarize, two independent approaches showed that the generated antibody AB-321 specifically recognized the mouse TRPM5 protein.

To test whether the TRPM5-specific antibody AB-321 is suitable for the detection of TRPM5 in rodent tissues we performed immunohistochemistry of paraffin sections of valate papillae (Figure 1C,D,E). In line with recent reports [7,8], AB-321 elicited a strong immunofluorescent signal in a subpopulation of TRCs in taste buds, while the surrounding tongue tissue was negative for TRPM5. TRPM5 immunoreactivity was blocked by the corresponding immunization peptide (Figure 1F). Notably, we only detected a TRPM5 immunofluorescent signal at the basolateral membrane of some TRC. In order to confirm this observation, we performed co-localization studies of TRPM5 and the lectin Ulex Europaeus Agglutinin (UEA), a membrane marker of all taste bud cells (Figure 1G–I) [32]. In fact, we found that TRPM5 is mainly localized at the basolateral cell surface of TRC and is absent in the gustatory pore, readily stained by UEA.

### Expression of TRPM5 in chemosensory organs

Due to its biophysical characteristics (activation mechanisms, fast kinetics of activation and inactivation, high temperature sensitivity) TRPM5 appears to be well suited to function in sensory cells. Therefore, we tested the hypothesis that TRPM5 may be present in sensory organs other than taste buds.

**Figure 2:**
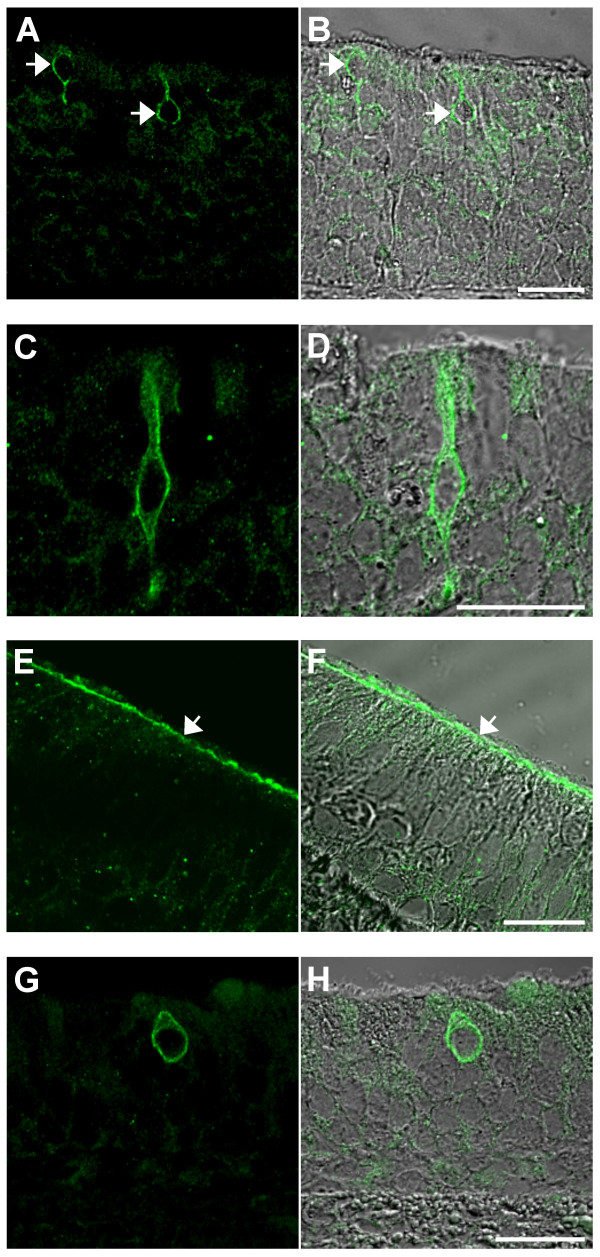
**TRPM5 expression in mouse main olfactory epithelium and the vomeronasal organ**. Confocal images of TRPM5-specific signals (left panels) and overlay with corresponding DIC images (right panel) are depicted. (**A-D**) Immunolocalization of TRPM5 in solitary cells of the main olfactory epithelium is shown at lower (indicated by the arrows in (A, B)) and higher magnifications (C, D). (**E-H**) Immunohistochemistry of TRPM5 in the VNO. (E, F) Localization of TRPM5 on the apical surface of sensory epithelia (indicated by the arrow). (G, H) Detection of TRPM5-enriched solitary cells in the non-sensory part of the VNO. Scale bars are 20 μm.

While we did not detect TRPM5 in the retina (data not shown), we obtained positive signals in chemosensory organs: the olfactory epithelium and the VNO (Figure 2). In the main olfactory epithelium, we detected TRPM5 in solitary epithelial cells whose nuclei were mainly localized superficially, at the level of the supporting cell layer (Figure 2A,B). Occasionally, few nuclei of TRPM5 immunopositive cells appeared in the basal part of the epithelium (data not shown). Like typical ciliated olfactory cells, TRPM5-expressing cells have elongated cell bodies that reach both the lumen and the basal membrane (Figure 2C,D).

In addition, we detected TRPM5 in sensory and non-sensory epithelia of the VNO. In the sensory epithelium, TRPM5 was localized at the apical part of the cell (Figure 2E,F), while in the non-sensory epithelium, TRPM5 was present in solitary immunoreactive cells scattered throughout the luminal part of the epithelium (Figure 2G,H). Thus, our data suggest that TRPM5 might play an as yet unappreciated physiological role in olfaction of odorants and pheromones.

### TRPM5 is enriched in brush cells of the respiratory system

**Figure 3:**
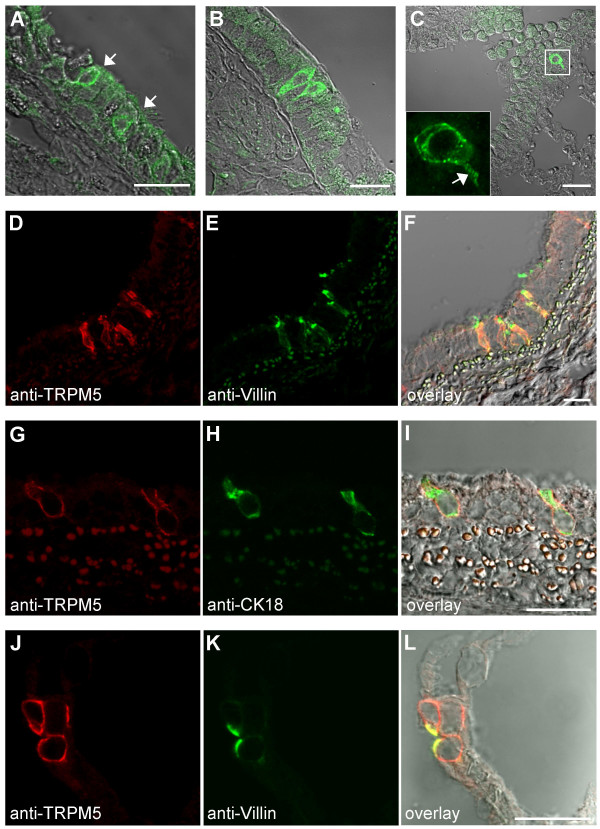
**TRPM5 is specifically expressed in brush cells of the respiratory system**. Detection of solitary TRPM5-expressing cells in the mouse respiratory epithelium of nose (indicated by arrows in (A)), trachea (B) and bronchus (C). Overlays of TRPM5 fluorescence with the corresponding DIC images are shown. The inset in (C) shows the fluorescent image of a TRPM5-positive cell with a basolateral extension (indicated by the arrow). (**D-L**) TRPM5-expressing cells express brush cell-specific marker proteins. Confocal images of TRPM5-specific signals (left panels), villin or CK18 (middle panels) and their overlays with corresponding DIC images (right panel) are shown. Note the co-localization of TRPM5 with villin (D-F) and CK18 (G-H) in rat trachea and TRPM5 with villin in rat alveolar epithelium (J-L). Scale bars are 20 μm.

**Figure 4:**
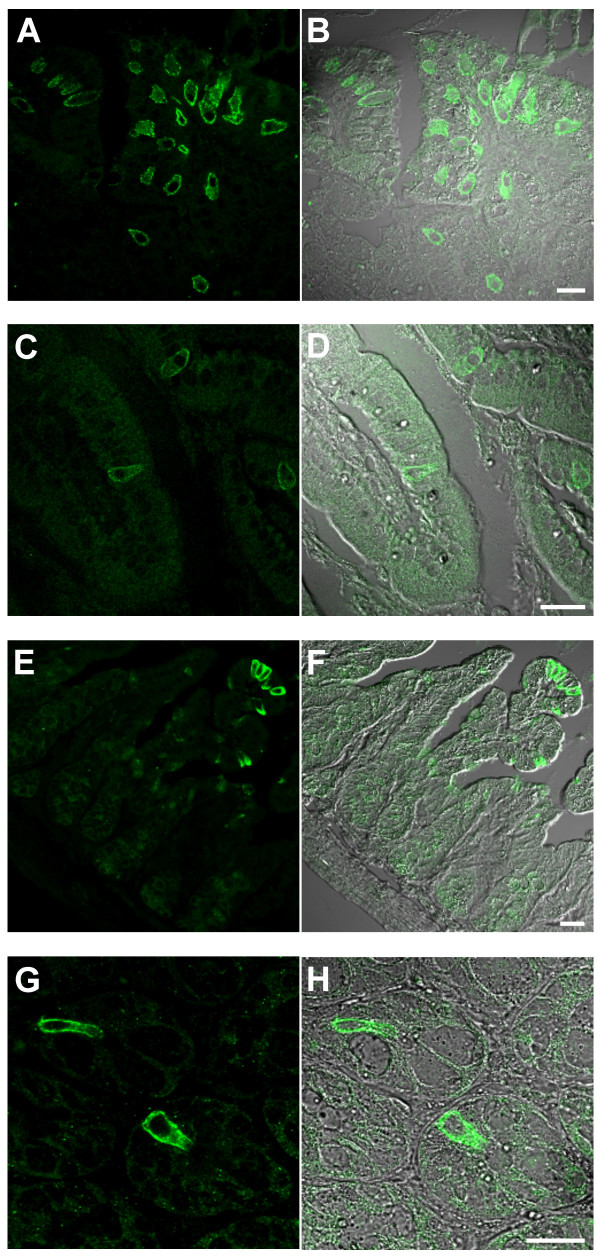
**Distribution of TRPM5-immunoreactive cells in the mouse gastrointestinal tract**. Confocal images of TRPM5-specific signals (left panel) and their overlay with the corresponding DIC images (right panel) are depicted. (**A, B**) TRPM5-expressing cells are abundant in the cardiac region of the stomach. In gut epithelia, TRPM5-enriched cells are detected in the duodenal villi (**C, D**), the ileum (**E, F**) and the colon (**G, H**). Scale bars are 20 μm.

**Figure 5:**
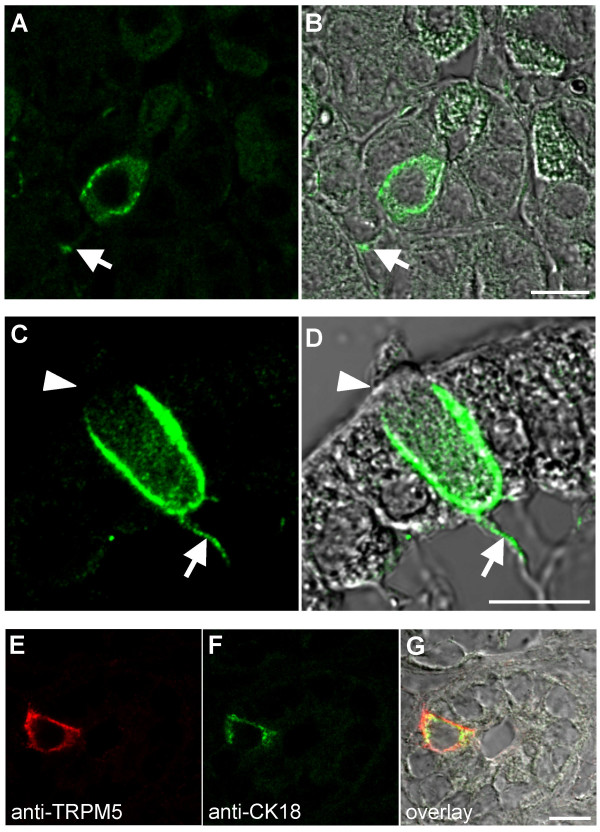
**TRPM5-expressing cells of the gastrointestinal tract have morphological and biochemical characteristics of brush cells**. (**A-D**) Morphological features of TRPM5-expressing cells. Confocal images of TRPM5-specific signals (left panels) and overlay with corresponding DIC images (right panels) are shown. The TRPM5-enriched cells in gastric glands of mouse stomach (A, B) and in mouse a duodenal villus (C, D) reveal a long basolateral processes (indicated by the arrows). Within duodenal epithelia (C, D), the TRPM5-immunoreactive cell contains a protrusion extending beyond the epithelial luminal border (indicated by an arrowhead). (**E-G**) Co-localization of TRPM5 with CK18 in intestinal glands of the rat duodenum. Confocal images of TRPM5 and CK18 specific signals (E, F) and their overlay with corresponding DIC images (G) are depicted. Scale bars are 10 μm.

By means of RT-PCR and Northern blot analysis, TRPM5 expression has been demonstrated in many tissues [33,34]. Therefore, we systematically searched rodent organs for TRPM5 protein expression by immunohistochemistry. TRPM5 could not be detected in liver, pancreas, kidney, spleen, lymph nodes, vessels, salivary glands, ovary, blood cells, fat cells, skeletal and smooth muscle (data not shown). We did, however, observe TRPM5 immunoreactive cells in the respiratory system (Figure 3) as well as in the gastrointestinal tract (Figures 4, 5).

In the respiratory system, TRPM5 expression was restricted to solitary epithelial cells of the nose, trachea and bronchus (Figure 3A–C). Occasionally, in some regions of the tracheal epithelium TRPM5-expressing cells appeared in clusters (Figure 3D–F). Remarkably, the apical surface of TRPM5-positive cells contacted the lumen, while the basolateral part bore a long extension (Figure 3C). Like in TRCs, TRPM5 in respiratory tract cells was restricted to the basolateral membrane (Figure 3D–I).

The distribution and morphological characteristics of these TRPM5-expressing cells were indicative of neuroendocrine cells, neuroendocrine bodies or brush cells [35-37]. In order to assign the TRPM5-expressing cells to one of these entities, we performed double immunostainings against TRPM5 and cell-specific markers.

Typical markers for neuroendocrine cells and bodies, i.e. chromogranin A, protein gene product 9, and calcitonin gene-related peptide [38] were found not to co-localize with TRPM5 (data not shown). It is well established that brush cells of the respiratory system and the gastrointestinal tract display unique morphological features not observed in any other epithelial cells [30,39,40]. First, their numerous apical microvilli are highly enriched in filaments which extend basally into the supranuclear area. Second, brush cells contain microvilli with corresponding rootlets of filaments extending from the lateral cell surface. Consequently, fluorescence microscopy enables the detection of intense immunoreactivity of villin in the apical as well as in the lateral regions of brush cells [41]. Another remarkable feature of brush cells is that their enrichment in villin is invariably accompanied by an intense immunoreactivity to cytokeratin 18 [42]. Therefore, we used antibodies specific for villin or anti-cytokeratin 18 (CK18), for co-immunostainings of these proteins and TRPM5 in tracheal tissue sections. We noted an invariable co-localization of these protein markers and TRPM5 in the same cells (Figure 3D–I) indicating that TRPM5-positive cells of the trachea are most probably brush cells. Rat alveolar epithelia contain solitary villin-positive cells considered to be brush cells [42]. Therefore, we tested whether these cells that we could detect with an anti-villin antibody, would also yield a positive TRPM5 staining. Figure 3J–L shows that TRPM5 and villin are invariably co-localized in solitary alveolar cells.

### Localization of TRPM5 in the gastrointestinal tract

Brush cells are known to be expressed not only in the respiratory system, but throughout the gastrointestinal tract, and Northern blot analysis indicates a considerable enrichment of TRPM5 mRNA in the intestine [7,43]. Thus, we extended our study to a detailed immunohistochemical analysis of the gut. Throughout the gastrointestinal tract, we detected solitary TRPM5-positive cells (Figure 4). In the stomach, TRPM5-enriched cells were readily seen in the principal part of the gastric glands, most abundantly in the cardiac region (Figure 4A,B). In the small intestine (Figure 4C,D) and colon (Figure 4G,H) scarce solitary TRPM5-positive cells were scattered throughout the epithelial sheet with a conspicuous enrichment in the villar region in the ileum (Figure 4E,F).

Similar to their counterparts in the tracheal epithelium, the TRPM5-positive cells in the gastrointestinal tract share morphological features with brush cells (Figure 5). Typically, these cells directly face the luminal surface, and long basal extensions reach the lamina propria (Figure 5A–D). Unlike the surrounding gut epithelial cells, TRPM5-positive cells display a conspicuous apical microvillar tuft reminiscent of brush cells (Figure 5C,D). Moreover, like in TRC and respiratory brush cells, TRPM5 expression sites were confined to the basolateral cell surface.

In order to determine whether or not the cells detected were *bona fide *brush cells, we performed immunohistochemical co-stainings of TRPM5-positive cells with the brush cell marker CK18 (Figure 5E–G). Most of the TRPM5-expressing cells displayed a clear co-localization with CK18. However, we found a minor CK18-negative fraction (~30%), either due to limited sensitivity, or because these cells represent a distinct subtype. Conversely, all cells staining positively for CK18 clearly expressed TRPM5.

To summarize, we found that TRPM5 is expressed throughout the gastrointestinal tract, specifically in solitary epithelial cells, which can be classified as brush cells based on cell distribution, morphological criteria and the presence of a specific marker protein.

## Discussion

Studies on TRP channels have significantly extended our knowledge of the molecular events underlying the sensation of temperature, mechanical force, smell and taste. Thus, TRPM5 was recently shown to be enriched in TRC and is essential for the perception of sweet, bitter and umami taste compounds [7,9,10]. However, it is well documented in the scientific literature that the expression pattern of TRPM5 is not restricted to taste buds. These findings prompted us to test the hypothesis that TRPM5 may play a broader functional role in chemosensation than initially suggested by experiments with TRPM5-deficient mice.

To this end, we raised and tested anti-TRPM5 antibodies whose staining pattern of taste receptor cells is fully congruent with previous work by other researchers [7,8]. However, performing a more detailed analysis, we observed in addition that the basolateral plasma membrane of TRC is readily stained, whereas the apical part, the gustatory pore, is spared – a principle applying to all other TRPM5 positive cells investigated with the notable exception of cells in the sensory epithelium of the VNO. These observations provide further insight into the function of TRPM5 in TRC. Most other TRP channels that are involved in sensory signaling are eo ipso receptors of extra- or intracellular messengers and are found at the exact location of primary signal detection. This notion holds true for both direct signal detectors like heat- or cold-sensing TRPs [44] as well as for TRPC channels that are activated by a receptor and G protein-dependent signaling cascade like TRP and TRPL in the insect rhabdomere [45], the TRPC2 channel expressed in the dendritic knob of rodent VNO [3] or TRPC6 expressed in microvillar cells of the main olfactory epithelium [46]. Our data suggest that TRPM5 is not directly involved in the process of taste recognition. Activation of TRPM5 may depolarize the basolateral plasma membrane resulting in the modulation of other voltage-dependent ion channels and subsequent neurotransmitter release. Alternatively, the TRPM5-mediated depolarization of the basolateral membrane could modulate the function of gap junctions, and, thus change the physiological status of neighboring cells [47-49].

Our studies with other sensory organs strongly indicate that in addition to taste perception TRPM5 is also involved in the processing of olfactory stimuli. Accordingly, we identified TRPM5 in two chemosensory organs, VNO and olfactory epithelia.

We detected TRPM5 in solitary cells within the non-sensory epithelium and apically in the sensory epithelium of the VNO. Interestingly, another member of the TRP gene family, the Ca^2+^-permeable TRPC2 is also present on the apical cell surface of the sensory epithelium of the VNO [3,4]. Thus, it is imaginable that in conjunction with TRPC2, TRPM5 is required for the detection of pheromones. In this context it should be recalled that TRPM5 cDNA was isolated from the VNO [12]. Furthermore, CAN channel activity with electrophysical properties similar to TRPM5 or TRPM4 was measured in freshly isolated VNO neurons [12,50]. Preliminary findings of our group using an anti-TRPM4 antibody illustrate that TRPM4 is highly expressed in the sensory epithelium of the VNO where it localizes to the apical region of sensory cells like TRPM5 (data not shown). These findings raise the possibility that in the VNO, TRPM4 and TRPM5 are co-expressed and co-localized and might therefore assemble to form heteromultimeric channels whose subcellular trafficking and functional properties could differ from either channel expressed alone in other cells.

In the olfactory epithelium, TRPM5 was found in scattered cells. The morphology and distribution of TRPM5-expressing cells are reminiscent of microvillar cells which are thought to be involved in sensing of odorants [46]. By means of immunohistochemical criteria, it was recently shown that microvillar cells can be classified into at least two subtypes, one of which was denoted brush cells [51] that were also found to be localized in the non-sensory epithelium of the VNO [52]. The physiological relevance of TRPM5 expression in the VNO and the olfactory epithelium still remains to be clarified by a meticulous analysis of odorant and pheromone sensing in TRPM5-deficient mice. While this manuscript was under review, Lin W. et al. [53] reported expression data similar to ours for TRPM5 in the main olfactory epithelium. Furthermore, these authors provided evidence for an involvement of TRPM5 in the sensing of semiochemicals, compatible with our hypothesis that TRPM5 may take part in chemosensation.

It was recently postulated that a population of specialized cells of the respiratory epithelium and gastrointestinal tract forming the so-called diffuse chemosensory system participates in the recognition and processing of diverse environmental cues [30,54]. This assumption was recently substantiated by the immunolocalization of signaling proteins such as Gα_gust_, T2R, PLCβ2 and the IP_3_III receptor in solitary intestinal epithelial cells and in cells of the respiratory system.)[23,25-29,55]. In accordance with these earlier findings, Bezençon et al. took advantage of TRPM5 promoter-based eGFP transgenic mice and identified GFP-positive cells in the gastrointestinal tract which partially co-express a set of taste signaling proteins mentioned above [56].

These observations are in line with our data demonstrating TRPM5 expression in scattered epithelial cells of the respiratory system and gastrointestinal tract. The cell body of TRPM5-enriched cells is bottle-shaped. The apical elongated narrow cell pole directly contacts the lumen and protrudes beyond the neighboring epithelial cells. On the basolateral cell pole, TRPM5-expressing cells have a long process contacting the lamina propria. Thus, the distribution and morphological characteristics of TRPM5-immunopositive cells conspicuously share numerous features with brush cells [30,40]. To identify the cellular origin of TRPM5-immunopositive cells, we resorted to double immunofluorescence staining using antibodies directed against villin or CK18, proteins known to be enriched in brush cells [41,42]. We observed an invariable co-localization of these marker proteins with TRPM5 in the respiratory system and a predominant co-localization in the duodenum.

Brush cells were initially defined by morphological criteria, and a clear biological role of this cell population still remains elusive. Hypotheses about these cells' functional role range from absorptive processes [57], mechanosensation [58], secretion [59-61] or a contribution to the local immunological defense barrier [62-66]. In the respiratory system and the gastrointestinal tract, brush cells may play a role in sensation of chemical compounds in the lumen [23,24,35,39,67-69]. The latter notion is derived from two salient observations: Firstly, brush cells display morphological features similar to TRC. Both cell types are characterized by an apical tuft of stiff microvilli with long rootlets into the cytoplasm lacking classical exocytotic vesicles or synapses [8,17,30,40]. Secondly, several signaling proteins pertinent to taste perception were identified in a subset of brush cells, including α-gustducin [23,24] and NO-synthase [70]. To our knowledge, TRPM5 is the first protein identified to be specifically expressed in brush cells of the gastrointestinal tract and the respiratory system. These results lend further support to the view that taste cells and brush cells share a common functional principle, i.e. chemosensation, and that brush cells are an important cellular correlate for chemosensation in the periphery.

## Conclusion

The abundant and specific expression of TRPM5 in taste receptor cells, the VNO and the olfactory epithelium is consistent with the assumption that TRPM5 has a common functional role in sensation of taste compounds, olfaction of odorants and pheromones. TRPM5 is the first protein identified to show high and invariable enrichment in brush cells of the gastrointestinal tract and the respiratory system. Our data are suggestive of brush cells as a specialized population of epithelial cells potentially involved in chemosensation and of TRPM5 as a characteristic molecular constituent of the sensory machinery of these cells.

## Methods

### Antibodies and chemicals

The following antibodies were used: mouse anti-villin (clone ID2C3, Immunotech, Coulter, Marseille, France), mouse anti-cytokeratin 18 (CK18) (clone CY-90, Sigma, St. Louis, USA), goat anti-rabbit IgG/horseradish peroxidase conjugate (Bio Rad, Munich, Germany), goat anti-rabbit IgG conjugated to Alexa488 (Molecular Probes, Leiden, Netherlands), fluorescein-isothiocyanate (FITC)-labeled donkey anti-mouse antibody f(ab')_2_-fragment (Dianova, Hamburg, Germany) and indocarbocyanin (Cy3)-labeled donkey anti-rabbit antibody (Chemicon, Hampshire, United Kingdom). The lectin Ulex Europaeus Agglutinin (UEA) conjugated to rhodamine was purchased from Vector laboratories (Burlingame, USA).

### Molecular biology, generation of TRPM5 specific polyclonal peptide-antibody and peptide control

To generate TRPM5 N-terminally fused to enhanced yellow fluorescent protein (YFP), the start codon was replaced by an EcoR1 restriction site by site-directed mutagenesis (XL QuickChange Mutagenesis kit, Stratagene, La Jolla, CA) followed by in-frame subcloning of eYFP as described before (online Supporting Methods, PNAS Chubanov *et al*. 2004).

Polyclonal TRPM5-specific antibodies were raised by immunization of rabbits with the following peptides coupled via the N-terminus to keyhole limpet hemocyanin (KLH): H_2_N-ARDREYLESGLPPSDT-COOH (AB-321), H_2_N-CSTHPLLLEDSPS-COOH (AB-391) and H_2_N-CAEHKREHLERDLPD-COOH (AB-392) (Standard immunization program, Eurogentec, Seraing, Belgium) and purified by peptide affinity chromatography. All three antibodies displayed specific binding to the TRPM5 protein in Western blot, immunocytochemistry and immunohistochemistry. Of the three antibodies raised, AB-321 displayed the highest sensitivity and was, therefore, used in all experiments shown. AB-391 and AB-392 were only used to verify the specificity of the AB-321- immunoreactivity.

As a control for the specificity of TRPM5 immunoreactivity, the AB-321 antibody was pre-cleared by an overnight incubation at 4°C with the agarose-immobilized immunization peptide. This procedure abolished TRPM5 immunoreactivity in all experiments reported here.

### Cell culture, Western blot analysis and immunocytochemistry on cells

Human embryonic kidney (HEK) 293 cells were cultured at 37°C and 5% CO_2_in Earle's MEM supplemented with 10% [v/v] fetal calf serum, 100 U/ml penicillin, 100 μg/ml streptomycin. For transient expression, cells were seeded on 35-mm dishes for Western blot analysis or on 25-mm glass coverslips for immunocytochemistry. 90% confluent cells were transfected with 1.5 μg of TRPM5 or YFP-TRPM5 cDNAs using the METAFECTENE transfection reagent (Biontex Laboratories, Munich, Germany). Cells were analyzed 24 h after transfection.

For Western blot analysis, transfected HEK 293 cell were washed twice in phosphate buffered saline (PBS), pH 7.4, lysed with 500 μl 2 × Laemmli-buffer at 65°C for 10 min. Subsequently, 20 μl of the lysates were subjected to SDS gel electrophoresis (7% polyacrylamide) and blotted onto nitrocellulose membranes (Hybond-C Extra, Amersham Biosciences, Freiburg, Germany). Non-specific binding was blocked by incubation of the membrane with 10% non-fat dry milk in PBS for 2 h at room temperature. Next, membranes were incubated with the AB-321 TRPM5-specific antibody (2 μg/ml in 5% BSA in PBS) overnight at 4°C. After washing in PBS (3 × 10 min), membranes were incubated with the secondary goat anti-rabbit IgG conjugated to horseradish peroxidase (1:5000 in 5% non-fat dry milk in PBS) for 1 h at room temperature. Subsequently, membranes were washed in PBS (3 × 10 min) and peroxidase activity was detected with an enhanced chemoluminescence detection reagent (ECL; Amersham Biosciences, Freiburg, Germany).

For immunocytochemistry, transfected HEK 293 cells were washed with PBS and fixed with 4% formaldehyde in PBS, pH 7.4 for 20 min. After washing with PBS (2 × 5 min), cells were incubated in 0.1% Triton X-100 in PBS for 20 min and then in blocking buffer (10% normal goat serum in PBS) for 1 h at room temperature. Subsequently, the AB-321 antibody (2 μg/ml) in 5% goat serum in PBS was applied overnight at 4°C. Cells were washed in PBS (3 × 10 min) and were incubated with 1 μg/ml secondary goat anti-rabbit IgG conjugated to Alexa488 in 5% goat serum in PBS for 1 h. After the final washing step, coverslips were placed on glass slides using a mounting medium.

### Immunohistochemistry on tissue sections

All experiments involving animals were approved by the local council on animal care. TRPM5 expression pattern and co-localization with UEA were examined in paraffin sections. BALBc mice and Wistar rats of both sexes were killed by cervical dislocation. Tissues (about 1 cm^2^) were dissected and fixed in 4% formaldehyde in PBS, pH 7.4 overnight. For paraffin embedding, the vacuum infiltration processor was used according to the instruction of Tissue-Tek^® ^(Vogel, Gießen, Germany). 1 μm sections were prepared using the microtome Accu-Cut^® ^SRM™ 200 (Sakura, Zoeterwoude, Netherlands) and dried on glass slides at 60°C for 2 h. Subsequently, sections were dewaxed in xylene (2 × 10 min), rehydrated in descending concentrations of ethanol in water (2 × 100%, 80%, 70% for 5 min) and finally rinsed in water for 5 min. For demasking of epitopes, the sections were incubated in 10 mM citrate-buffer, pH 6 for 10 min at 95°C and then washed in PBS for 10 min at room temperature. Next, tissue sections were blocked in 10% goat serum in PBS for 1 h and incubated with the AB-321 TRPM5 antibody in 5% goat serum in PBS at 4°C overnight. After washing in PBS (3 × 10 min), sections were incubated with 1 μg/ml secondary goat anti-rabbit IgG conjugated to Alexa488 in 5% goat serum in PBS for 1 h. Subsequently, sections were washed in PBS (3 × 10 min), mounted and examined by confocal microscopy. For the double labeling with the TRPM5 antibody and UEA, 10 μM rhodamine-conjugated UEA was added to the secondary antibody solution.

Co-localizations of TRPM5 with villin or CK18 were studied on cryosections of rat tissues. To this end, Wistar rats were sacrificed, and the required tissues were dissected, shock-frozen in liquid-nitrogen-cooled melting 2-methylbutane and embedded in O.C.T compound (Sakura, Zoeterwoude, The Netherlands). 10 μm thin sections were prepared and air-dried. For double immunostainings against TRPM5 and villin, sections were fixed in 2% [w/v] paraformaldehyde in 0.2 M sodium phosphate-buffer (pH 7.4) for 20 min at room temperature. After washing in PBS (2 × 10 min) and in water (1 × 10 min), sections were air dried for 1 h and then blocked with 10% horse-serum/0.5 % Tween/0.1% BSA in PBS for 1 h. The mixture of rabbit anti-TRPM5 and mouse anti-villin antibody (1:50) was applied overnight at 4°C. For co-localization of TRPM5 and CK18, sections from rat tissues were fixed in acetone for 10 min at -20°C, air dried for 1 h and blocked with 5% BSA and 5% normal goat serum in PBS for 1 h. Next, a mixture of primary antibodies was used: the rabbit anti-TRPM5 and mouse anti-CK18 antibody (1:800) in PBS overnight at 4°C.

After incubation with the primary antibodies, sections were washed in PBS (3 × 10 min). Subsequently, a mixture of donkey anti-rabbit antibodies conjugated with Cy3 (1:2000) and donkey anti-mouse antibodies conjugated with FITC (1:200) were applied for 1 h at room temperature. After washing in PBS (3 × 10 min), sections were embedded and examined by confocal microscopy (see below).

### Confocal microscopy

Confocal and differential interference contrast (DIC) images of cells or tissues were obtained with a LSM 510 META confocal laser scanning microscope (Carl Zeiss, Jena, Germany). For the detection of Alexa 488 fluorescence, we used a Plan-Apochromat x63/1.4 oil objective, the 488 nm excitation wavelength of an argon laser, and a 505–530 nm band-pass filter. The pinhole diameter was set to yield optical sections of ~0.6 μm. In co-localization experiments, confocal images of FITC or Alexa488 and Cy3 or rhodamine fluorescence were acquired using Plan-Apochromat x63/1.4 oil objective, 488 nm (argon laser) and 545 nm excitation wavelengths (helium-neon laser), the 505–530 nm band-pass filter and a 560 nm long-pass filter. The pinhole diameter was set to yield optical sections of ~0.6 μm. Acquired DIC and confocal images were analyzed and combined using the LSM 510 META software (Carl Zeiss, Jena, Germany).

## Authors' contributions

SK, VC, PK, WK, TH, TG designed and analyzed the experiments. SK GK, PK and WK performed immunostainings. VC carried out the molecular biology and designed the TRPM5 and TRPM4 antisera. SK, TH, VC, and TG wrote the manuscript. All authors read and approved the final manuscript.
